# Effects of autologous platelet-rich plasma coated sutures on intestinal anastomotic healing in rabbits

**DOI:** 10.1016/j.heliyon.2019.e02713

**Published:** 2019-11-28

**Authors:** Mousa Daradka, Mira M. Alardah, Zuhair Bani Ismail

**Affiliations:** Faculty of Veterinary Medicine, Jordan University of Science and Technology, Irbid, 22110, Jordan

**Keywords:** Biochemistry, Zoology, Dehiscence, Growth factors, Intestinal surgery

## Abstract

The objective of this study was to investigate the intestinal anastomotic healing enhancing effect of platelets-rich plasma (PRP) using PRP-coated sutures in a rabbit model. A total of 30 mature male rabbits were divided into 3 groups (10 rabbits per group). Group 1 received uncoated sutures, group 2 received sodium acetate-coated sutures, and group 3 received PRP-coated sutures. Polyglactin 910 (Vicryl, USA), size 4-0 was used in all groups. Five rabbits of each group were euthanized on day 3 following the surgery while the remaining 5 rabbits were euthanized on day 10. Gross evaluation of the anastomotic site in PRP-coated sutures group demonstrated significantly (P < 0.05) lower adhesion formation scores on both days 3 and 10 of the study while in the control groups, evidence of leakage at the anastomotic site was present along with signs of haemorrhage and local inflammation. On day 10 in the control groups, there were strands of strong adhesions between the ileum, colon and cecum with large amount of fibrin deposited at the site of the anastomosis. Tissues of the anastomotic site revealed a significant level of hydroxyproline on day 10 in PRP-coated sutures group compared with control groups. Histopathological evaluation revealed significantly (P < 0.05) less inflammatory infiltration, and more angiogenesis and collagen deposition on day 10 in PRP-coated sutures group compared to the control groups. Results of this study clearly indicate promising healing enhancing effects of using PRP-coated sutures at intestinal anastomotic site with little to no obvious disadvantages.

## 1 Introduction

Poor wound healing at intestinal anastomotic sites could lead to severe and life-threatening complications, additional surgeries, increased hospital stay and cost, discomfort and increased mortality (Kakudo et al., 2008; Pinto et al., 2016; Garg and Manchanda, 2017; Giusto et al., 2017; Shen et al., 2017). Indeed, despite today's medical advances in surgical techniques and postoperative management, the percentages of gastrointestinal anastomotic complications have still not been decreased to a negligible level (Fernández et al., 2017). Hence, novel means of evading anastomotic complications are believed essential and much anticipated.

Wound healing is dynamic and requires several coordinated and integrated physiological regenerative processes (Etulain et al., 2018). Several local and systemic factors can result in wound healing disruption leading to increased pain, suffering and sometimes life-threatening complications (Etulain et al., 2018). Fortunately, there are many techniques such as tissue transplantation, gene therapy, growth factors, and stem cell therapy that can be used to prevent wound healing disruption and or promote healing and repair of damaged tissues (Etulain et al., 2018). Substantial evidence suggests that platelet-rich plasma (PRP) enhances wound healing (Akhundov et al., 2012; Alsousou et al., 2013; Jain and Gulati, 2016). Activated platelets are rich with a triad of growth factors, which are biologically active polypeptides that influence growth, metabolisms and differentiation of target cells via activation of specific receptors (Lee et al., 2011). The platelet derived growth factor (PDGF), transforming growth factor–β (TGF-β), vascular endothelial growth factor (VEGF), epidermal growth factor (EGF), and adhesive proteins – fibrin, fibronectin, and vitronectin all have been recognized in the intricate wound healing process (Eppley et al., 2004; Lee et al., 2011; Jain and Gulati, 2016; Alves and Grimalt, 2018).

It is believed that PRP influences wound healing by stimulating tissue repair mechanisms, enhancing tissue healing and regeneration including bone, cartilage, tendon and muscle at various levels with minimal adverse effects (Albanese et al., 2013; Alsousou et al., 2013; Rah et al., 2017; Lang et al., 2018; Tambella et al., 2018a,b). Indeed, PRP has been used to enhance the healing process of various types of wounds (Kim et al., 2009; Alsousou et al., 2013; Picard et al., 2015; Alishahi et al., 2014; Tambella et al., 2014; Martinez-Zapata et al., 2016; Burgos-Alonso et al., 2018; Tambella et al., 2018a,b; Moneib et al., 2018), orthopaedics (Alsousou et al., 2013; Samy, 2016; Faillace et al., 2017; Marcazzan et al., 2018; Huang et al., 2019), ophthalmology (Ronci et al., 2015; Wróbel-Dudzińska et al., 2018; Alio et al., 2018), and dentistry (Del Fabbro et al., 2017; Tabrizi et al., 2018; Bhujbal et al., 2018; Saleem et al., 2018).

The hypothesis here is that wound healing at the intestinal anastomotic site can be enhanced by incorporating PRP into the sutures being used to close the anastomosis with no adverse effects and minimum adhesion formation. Therefore, this study was carried out to evaluate the intestinal anastomotic healing enhancing effects of PRP using PRP-coated sutures in a rabbit model.

## 2 Materials and methods

### 2.1 Animals

The study protocol was reviewed and approved by the Institutional Animal Care and Use Committee of Jordan University of Science and Technology (ACUC-JUST). In compliance with ACUC-JUST recommendation to reduce the number of animal subjects in research projects to a minimum, 30 mature male mixed-breed rabbits (weighing 2.0 ± 0.5 kg) were used in the study. Animals were housed in individual cages and were allowed free access to water and pelleted diet (Cuni-Elite, Deli Nature, Beligum).

### 2.2 Platelet rich-plasma harvesting

Approximately, 10 ml of autologous whole blood was collected aseptically via Jugular vein puncture from animals using vacutainer needles and glass blood tubes previously treated with 20 mM sodium citrate (Biomet, USA) as an anticoagulant. From this, 0.5 ml was used to determine original platelet count using an electronic cell counter (ABC Hematology Analyzer, Scil Animal Care Company, USA). The remaining 9.5 ml of blood were used to isolate PRP. The PRP was isolated immediately by centrifugation at 200 x g at 22 °C for 10 min. Then, the uppermost layer was transferred with a sterile pipette to another 10 ml centrifuge tube and re-centrifuged at 400 x g and 22 °C for another 10 min. About 0.5–1 ml of PRP was pipetted from the bottom of the tube (Landesberg et al., 2005). In this study, sodium citrate was used to suspend the platelet pellet instead of platelet poor plasma in order to make sure no plasma related growth factors are present that may interfere with the study results (Perazzi et al., 2013). The concentration of the platelet that was used to dip the sutures was approximately 1×10^6^ platelets/microL (Mazzucco et al., 2012).

### 2.3 Coating of the suture material

Polyglactin 910 suture materials (Vicryl; Ethicon, USA), size usp 4-0 and atraumatic needle were used to suture the anastomotic sites in all rabbit groups. The first set of sutures were not coated and used as it is (None-coated sutures). The second set of sutures was coated with 20 mM sodium citrate alone (SC- coated sutures). The third set of sutures were coated using 1 ml of PRP in sodium citrate (platelet count approximately 5×10^6^/ml) (PRP-coated sutures) using a dip-coating process (Dines et al., 2007). Briefly, sutures were treated with 70% ethanol, then the sutures were dipped in sodium acetate aseptically in a sterile laminar flow hood, with or without PRP for 30 min and then left to air-dry at room temperature.

### 2.4 Scanning electron microscopy of sutures

To visualize PRP on the coated and uncoated sutures, three suture samples were used for the scanning electron microscopy study (None-coated, SC-coated and PRP-coated). Suture samples were cut into 2mm sections, allowed to air-dry at room temperature and coated in gold Quorum Q150R (Quorum Technologies Ltd, UK). Samples were then analysed using Quanta FEG 450 (Thermo Fisher Scientific, USA).

### 2.5 TGF-β1 and PDGFA concentrations

The transforming growth factor-β1 and platelet-derived growth factor-A concentrations released from PRP-coated sutures into culture media at 4 time points (1, 2, 24 and 48 hours) were determined using commercially available ELISA kits according to manufacturer's instructions (Mybiosource, California, San Diego, USA) and (Abbexa, Cambridge, UK), respectively according to the manufacturer's instructions.

### 2.6 Tensile strength of suture

The biomechanical strength of coated and uncoated suture materials was tested by a load to failure test using a computer controlled electromechanical material testing machine (China Wdw-20 Universal Testing Machine, China).

### 2.7 Intestinal anastomosis

The rabbits were subjected to general anaesthesia using intramuscular injection of 2% xylazine (Xylaject, Egypt) at 5 mg/kg body weight followed by intramuscular injection of ketamine hydrochloride (Ketaset; Zoetis, USA) at 40 mg/kg body weight.

**Fig. 1:**
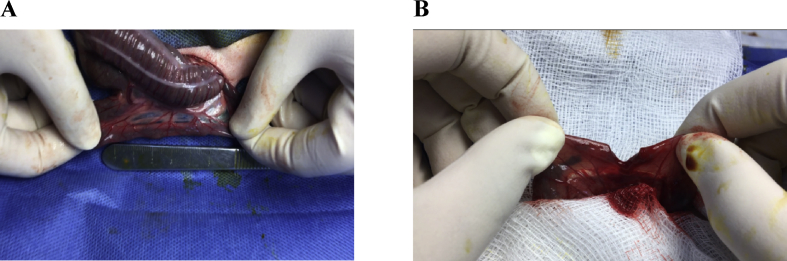
A segment of the distal ileum is identified and exteriorized for intestinal enterectomy (A) Enterectomy site approximately 5 cm distal to the ileocecal junction (B) Enterectomy was performed at the distal ileum.

Ventral midline laparotomy was performed under aseptic conditions according to the previously described procedure (Testini et al., 1998). The abdominal cavity was thoroughly explored to exclude abnormal conditions involving the gastrointestinal tract including adhesions and peritonitis. The ileocecal junction was then located and identified. A segment of the terminal ileum, at approximately 5 cm cranial to the ileocecal junction was identified and exteriorized (Fig. 1a). After gently evacuating the intestinal contents using non-traumatic forceps, the ileum was completely transected (Fig. 1b). An end-to-end anastomosis was performed using approximating suture pattern using none-coated sutures (group 1, n = 10), SC-coated sutures (group 2, n = 10), and PRP-coated sutures (group 3, n = 10). The anastomosis was tested for leaks and luminal continuity prior to layered closure of the abdominal wall. The abdominal wall was closed using size usp 4-0 polyglactin 910 suture material and continuous pattern. The rabbits were allowed to recover from anesthesia under close observation.

### 2.8 Clinical evaluation

Follow-up observation was carried out for 24h post-operatively. The rectal temperature was obtained using a digital thermometer once every day for the first 48 hours and then once on day 3 and day 7 post-operatively. The body weight was obtained using a digital scale on day 3 and again on day 7 after surgery.

### 2.9 Necropsy and gross evaluation

Five rabbits of each group were euthanized on day 3 following the surgery while the remaining 5 rabbits were euthanized on day 10 following the surgery. Euthanasia was performed by exsanguination under anesthesia using intramuscular injection of xylazine (5 mg/kg) and ketamine (40 mg/kg).

All rabbits were subjected to thorough and complete necropsy. Close gross examination of the anastomotic site, ilium, cecum, omentum and abdominal cavity was followed and any signs of inflammation, haemorrhage, fibrin deposition, adhesion formation or abscess formation were noted. The whole ileum was then excised and observed for adhesion formation. The adhesion was given scores of 0–3 according to previously described methods (Van der Ham et al., 1991). A value of 0 was assigned if there were no adhesions; value of 1 if there was minimum amount of adhesions between the anastomotic site and omentum; value of 2: if there was moderate amount of adhesions between anastomotic site and omentum and a value of 3 if there was extensive adhesions between the anastomotic site and omentum or abscess formation.

### 2.10 Biomechanical evaluation

The strength of each anastomosis was assessed by measuring of bursting pressure on day 3 following the surgery according to previously published methods (Martins et al., 2013). Briefly, the intestinal segment at the anastomotic site was progressively inflated with air by a catheter while an air pressure gauge was adapted to a second catheter, with the intent of measuring the rupture pressure of the suture line. The values of rupture, in mmHg, were recorded for each group.

### 2.11 Hydroxyproline measurement

Hydroxyproline levels (μg hydroxyproline/mg of tissue) in excised tissues from the anastomotic site were analysed on days 3 and 7 of the experiment using spectrophotometer (Hitachi, Japan) according to previously published methods (Ignateva et al., 2007). Briefly, 5 mm tissues were dried in a hot air oven at 60 °C to constant weight and were hydrolysed in 6 N HCl for 4 h at 130 °C. The hydrolysates were then neutralized to pH 7.0 and were subjected to Chloramine-T oxidation for 20 min. After 5 min, the reaction was terminated by the addition of 0.4 M perchloric acid and developed colour with Ehrlich reagent at 60 °C.

### 2.12 Histopathological evaluation

The anastomotic site was opened longitudinally and a 10 mm segment around the suture line was collected and placed in 10% buffered formalin for histopathology. Fixed tissue samples were embedded in paraffin, cut into 5 mm sections and stained using hematoxylin and eosin (HE) and Masson trichrome. Three different sections were taken from each subject and examined using light microscope at X40. The inflammatory cell infiltration, fibroblast proliferation and capillary vascular proliferation were scored using a scale of 0–4 according to previously published methods (Ceran et al., 2013). Histopathological evaluation was performed by one pathologist who was blinded to group assignments.

### 2.13 Statistical analysis

Statistical analysis was performed using SPSS software (IBM SPSS version 23). Between group comparisons were made using Kruskal-Wallis test followed by Mann–Whitney test. Chi-square test was used to compare the groups in terms of tensile strength, hydroxyproline levels, and histopathological scores. Values were considered statistically significant at P value <0.05.

## 3 Results

### 3.1 Clinical evaluation

All rabbits survived to the end of the study without showing any abnormal signs. There were slight increase in rectal temperature during the first 48 hours but temperature returned to normal on day 3 and remained normal through day 7. The body weight of the rabbits did not change significantly over the course of the experiment.

### 3.2 Platelets and PRP harvesting

The original blood samples from all rabbits contained on average 350 ± 150 × 10^3^ platelets/microL. Each 10 ml of whole blood produced about 0.5–1 ml of PRP with an average platelet count of about 6 ± 1.3×10^8^/microL.

### 3.3 Scanning electron microscopy of coated sutures

**Fig. 2:**
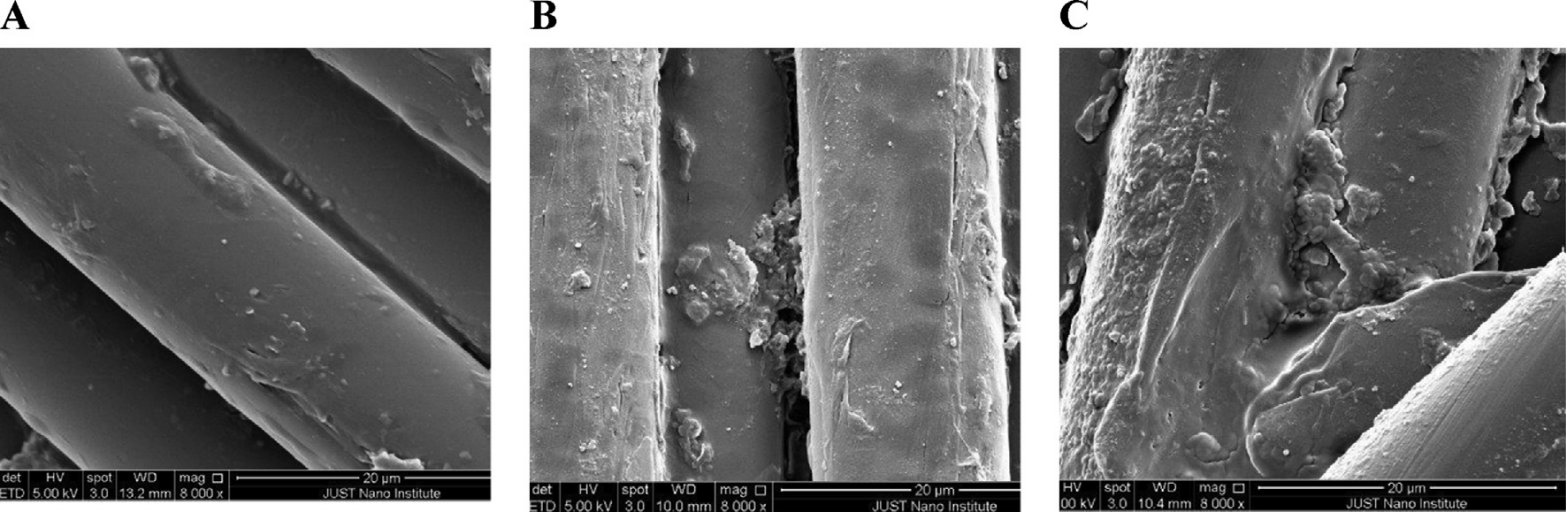
(A) Scanning electron microscopic images of PRP-uncoated sutures (B) Sodium citrate-coated sutures (C) PRP-coated sutures. In PRP-coated sutures, the images showed the intact nature of the coated sutures with aggregates pf platelets on its shafts and surface (Magnification 8000 X).

Scanning electron microscopic examination of PRP-coated and uncoated sutures are represented in Fig. 2. SEM images showed that coated sutures were intact similar to the uncoated sutures. Large number of platelets could be seen aggregated on the shafts and surfaces of coated sutures.

### 3.4 TGF- β1 and PDGFA of PRP-coated sutures

The concentrations (pg/ml) of platelet-derived growth factor subunit A (PDGFA) and transforming growth factor- β1 (TGF- β1) released from PRP-coated sutures at different time points are present in Table 1. There was a significant (P < 0.05) amount of TGF- β1 released from the PRP-coated sutures to the media within 1 h but this amount was back to base line level within 2 h and remained at base level at 48 hours. The amount of PDGFA was not significantly changes at any time point.

**Table 1:** Mean ± SD of transforming growth factor- β1 and platelet-derived growth factor subunit A (PDGFA) released from PRP-coated sutures at 4 different time points (pg/ml).

Time	TGF- β1	PDGFA
1 h	320 ± 32*	46 ± 6
2 h	260 ± 68	47 ± 5
24 h	276 ± 67	47 ± 5
48 h	237 ± 42	45 ± 7

*(P < 0.05).

### 3.5 Tensile strength of PRP-coated sutures

There was no significant differences (P = 0.211) in tensile strength between the PRP-coated and uncoated suture materials in terms of load to failure and deformity. The mean tensile strength in uncoated sutures, SC-coated sutures and PRP-coated sutures were 54.5 (±7.5), 55.6 (±10.2), and 60.2 (±5.5), respectively.

### 3.6 Necropsy findings and gross evaluation of the anastomotic site

**Fig. 3:**
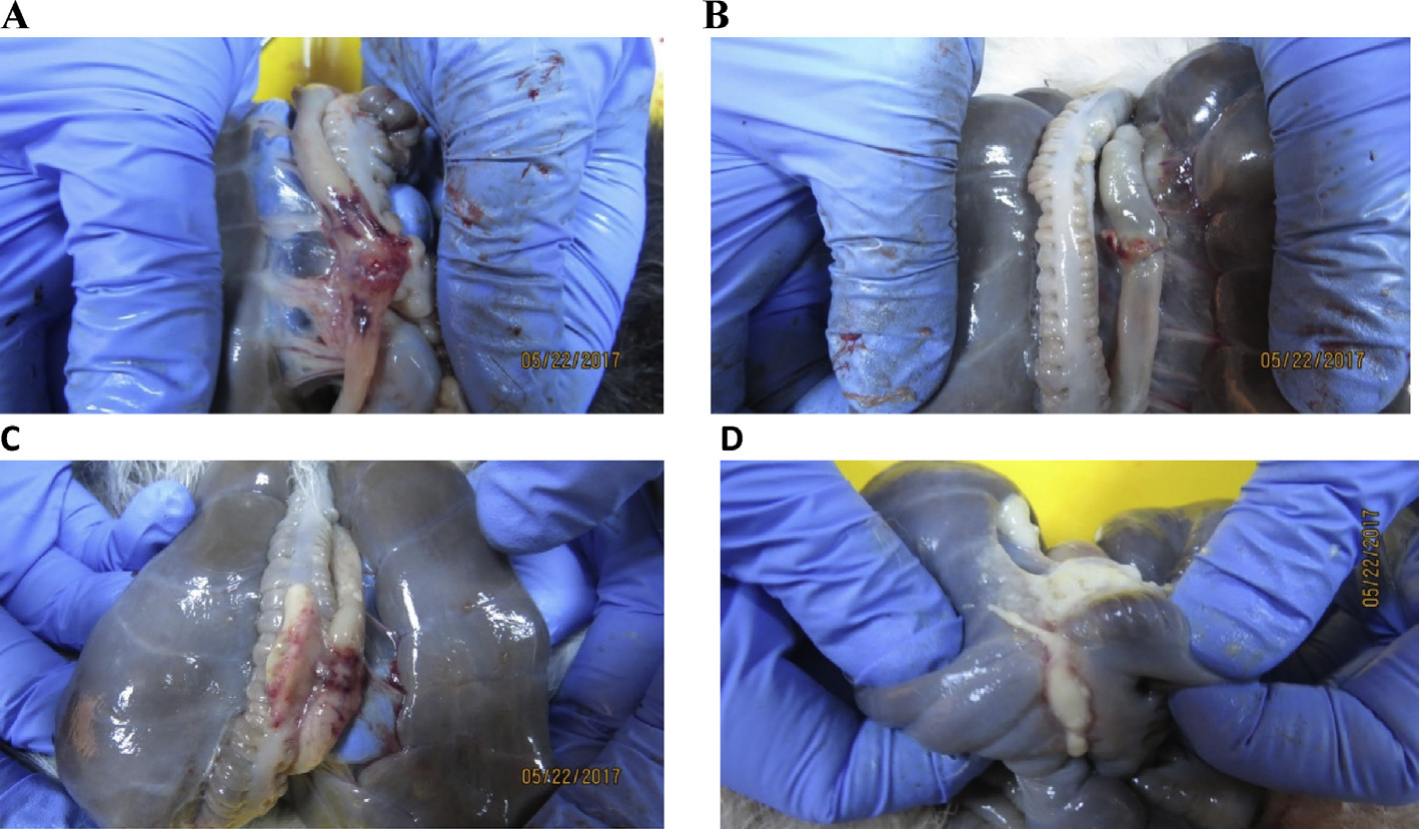
(A) The anastomotic site in the PRP-uncoated sutures group showing signs of haemorrhage and local inflammation (day 3) (B) The anastomotic site in PRP-coated group showing healing of surgical site with no leakage evident, no adhesions formation and no signs of inflammation (day 3) (C) The anastomotic site in the PRP-uncoated sutures group showing strong adhesion formation between the ileum, colon and cecum with large amount of fibrin deposited at the site of anastomosis (day 10) (D) The anastomotic site in PRP-coated group showing healing of surgical site with minimum adhesion formation and no signs of inflammation (day 10).

There were no overt signs of inflammation, haemorrhage or abscess formation in the abdominal cavity in any of the rabbits. Grossly, adhesion scores at the anastomotic sites were significantly (P < 0.05) lower in PRP-coated suture group (2 rabbits out of 10) on both days 3 and 10 of the study compared to PRP-uncoated (6 rabbits out of 10) and SC-coated groups (7 rabbits out of 10). In the PRP-uncoated suture groups, although adhesions were not apparent on day 3, evidence of leakage at the anastomotic site was present along with signs of haemorrhage and local inflammation in 3 rabbits (Fig. 3). On day 10, strong adhesions in 3 rabbits could be appreciated between the ileum, colon and cecum with large amount of fibrin deposited at the site of anastomosis in the PRP-uncoated suture groups (Fig. 3).

### 3.7 Hydroxyproline levels

Hydroxyproline tissue (μg hydroxyproline/mg of tissue) levels at the anastomotic sites showed no significant differences between groups on day 3 (Table 2). However, on day 10, the hydroxyproline levels in tissues obtained from anastomotic sites sutured with PRP-coated sutures were significantly elevated when compared to hydroxyproline tissue levels from the anastomotic sites sutured with uncoated sutures.

**Table 2:** Means ± SD of hydroxyproline tissue (μg hydroxyproline/mg of tissue) levels at the anastomotic sites sutured using PRP-uncoated, sodium citrate-coated, and PRP-coated suture materials.

Groups	Day 3	Day 10
PRP-uncoated sutures	0.36 ± 0.06	0.47 ± 0.13
Sodium citrate-coated sutures	0.37 ± 0.16	0.52 ± 0.07
PRP-coated sutures	0.34 ± 0.1	0.76 ± 0.1*

*P < 0.05.

### 3.8 Histopathological evaluation

**Fig. 4:**
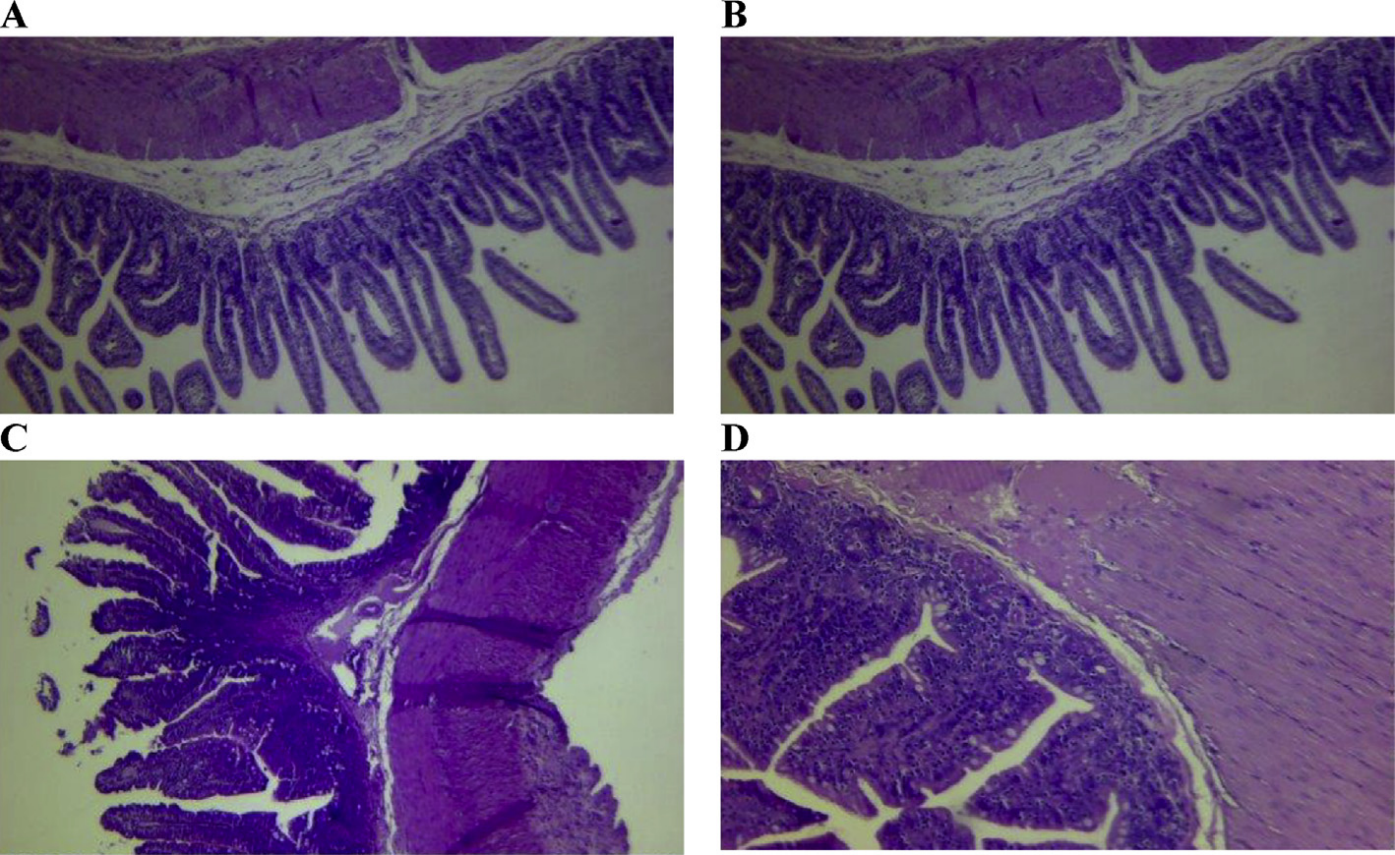
(A) Haematoxylin and eosin stained section of the anstomoyic site in control group at day 3 (40x magnification) showing the villi with normal appearance, edema in the submucosa and between the muscularis and the serosa (B) Haematoxylin and eosin stained section of the anstomoyic site in PRP-coated group at day 3 (40x magnification) showing slight submucosal edema with mimimal inflammatory infiltration (C) Haematoxylin and eosin stained of the anstomoyic site in control group at day 10 (40x magnification) shopwing the villi with normal appearance, edema in the submucosa and between the muscularis and the serosa (D) Haematoxylin and eosin stained section of the anstomoyic site in PRP-coated suture group at day 10 showing minmal inflammatory infiltration (40x magnification).

On day 3, there were no significant differences found between groups in terms of inflammatory cellular infiltration, angiogenesis, and collagen deposition (Fig. 4). However, on day 10, PRP-coated suture group had significantly (P < 0.05) less inflammatory infiltration, and more angiogenesis and collagen deposition compared to uncoated sutures groups.

## 4 Discussion

Platelets play a vital role in the first 72 hours following injury. At the site of injury, platelets release several growth factors that mediate the healing process. The use of autologous PRP is considered promising advance in gastrointestinal surgery (Sánchez-González et al., 2012; Giusto et al., 2017). To the best of our knowledge, this is the first study that investigated the effects of PRP on the healing of intestinal anastomosis in rabbits using coated sutures as a vehicle. Perioperative and postoperative clinical parameters such as body weight and body temperature in this study showed no significant changes between the groups, indicating the absence of any signs of anastomotic leakage or surgical complications. Although it is not possible to assess how much of the observed inflammatory reaction in the rabbits was because of the braided sutures that were used to perform the anastomosis in this study, we believe that this could have little effect on the final results of the study. Nevertheless, we recommended to use monofilament sutures in the anastomosis in future studies to overcome this limitation.

There were no significant differences in tensile strength between the PRP-coated and non-coated sutures which indicates that the coating process did not increase or decrease the strength of the sutures. These results are in agreement with others who reported a significant release of growth factors from PRP-coated sutures for up to 7 days after coating (Fufa et al., 2011). It has been suggested that coating duration and coating technique have a significant effect on the amount of growth factor released from PRP-coated sutures (Fufa et al., 2011).

Anastomotic bursting pressure and breaking strength are valuable standards for evaluating anastomotic strength and stability during healing (Yol et al., 2008; Yamaguchi et al., 2012). Bursting pressure is believed to be more exact measure as it reveals the physiologic strain in intestinal tissue (Yol et al., 2008; Yamaguchi et al., 2012). The outcomes acquired from the current study showed a significant rise in bursting pressure in the PRP-treated samples which was in agreement to previous findings using colonic anastomosis and jejunal anastomosis models in Sprague Dawley rats (Yol et al., 2008; Yamaguchi et al., 2012). Throughout the proliferative phase, augmented neovascularisation, fibroblast proliferation, and collagen deposition should be observed accompanied by decreased inflammation. Our results are also in accordance with those of Fresno et al. (2010). However, Fresno et al. (2010) concealed the anastomotic sites with omentum, leading to increased fibrosis and thus increased healing that could be reflected as biased. The omentum was not positioned on the anastomosis site in this study in order to evade this type of partiality.

Hydroxyproline is an exclusive collagen amino acid (Yol et al., 2008). Its presence in the anastomotic site indicates concurrent collagen synthesis and faster wound healing (Yol et al., 2008). This molecule is believed to intermediate wound healing by promoting collagen synthesis, deposition, and maturation (Yol et al., 2008). In this study, additional evidence reinforcing our assumption that PRP-sutures enhanced anastomitic healing is the statistically increased tissue hydroxyproline levels in the PRP-treated group compared to the control groups.

Histopathological examination of the anastomotic site showed increased fibroblastic proliferation and collagen creation (mainly in the serosal layer), increased neovascularization, inflammatory cell infiltration and an increased thickness of the intestinal wall with high presence of granulation tissue were on PRP-treated animals at 3 and 10 days postoperatively. These results are in agreement with those reported previously in rats (Yol et al., 2008). It was suggested previously that growth factors in PRP are responsible for better anastomotic strength and wound healing (Yol et al., 2008).

This study clearly exhibits the potential of coating sutures with PRP as a mean of accelerating healing by increasing granulation tissue and fibrosis with little to no obvious disadvantages. Further studies could be designed in the future to evaluate the clinical application of PRP in patients who undertake gastrointestinal anastomosis under impaired conditions for wound healing.

## Declarations

### Author contribution statement

Mousa Daradka: Conceived and designed the experiments.

Mira M. Alardah: Performed the experiments.

Zuhair Bani Ismail: Analyzed and interpreted the data; Wrote the paper.

### Funding statement

This work was supported by the Deanship of Research at Jordan University of Science and Technology.

### Competing interest statement

The authors declare no conflict of interest.

### Additional information

No additional information is available for this paper.
